# Psychological distress associated with the COVID-19 pandemic and suppression measures during the first wave in Belgium

**DOI:** 10.1186/s12888-021-03109-1

**Published:** 2021-02-18

**Authors:** Vincent Lorant, Pierre Smith, Kris Van den Broeck, Pablo Nicaise

**Affiliations:** 1 Institute of Health and Society (IRSS), Université Catholique de Louvain, Brussels, Belgium; 2 Family and Population Health (FAMPOP) & Collaborative Antwerp Psychiatry Research Institute (CAPRI),Faculty of Medicine and Health Sciences, University of Antwerp, Antwerp, Belgium

## Abstract

**Background:**

The COVID-19 pandemic and subsequent suppression measures have had health and social implications for billions of individuals. The aim of this paper is to investigate the risk of psychological distress associated with the COVID-19 pandemic and suppression measures during the early days of the lockdown. We compared the level of psychological distress at the beginning of that period with a pre-pandemic health survey and assessed the psychological effects of exposure to the COVID-19 pandemic and changes in social activity and support.

**Methods:**

An online survey was distributed to the general population in Belgium 3 days after the beginning of the lockdown. 20,792 respondents participated. The psychological distress of the population was measured using the GHQ-12 scale. Social activities and support were assessed using the Social Participation Measure, the Short Loneliness Scale, and the Oslo Social Support Scale. An index of subjective exposure to the COVID-19 pandemic was constructed, as well as a measure of change in occupational status. Measurements were compared to a representative sample of individuals extracted from the Belgian Health Interview Survey of 2018. Bootstrapping was performed and analyses were reweighted to match the Belgian population in order to control for survey selection bias.

**Results:**

Half of the respondents reported psychological distress in the early days of the lockdown. A longer period of confinement was associated with higher risk of distress. Women and younger age groups were more at risk than men and older age groups, as were respondents who had been exposed to COVID-19. Changes in occupational status and a decrease in social activity and support also increased the risk of psychological distress. Comparing the results with those of the 2018 Belgian Health Interview shows that the early period of the lockdown corresponded to a 2.3-fold increase in psychological distress (95% CI: 2.16–2.45).

**Conclusions:**

Psychological distress is associated with the consequences of the COVID-19 pandemic and suppression measures. The association is measurable from the very earliest days of confinement and it affected specific at-risk groups. Authorities should consider ways of limiting the effect of confinement on the mental and social health of the population and developing strategies to mitigate the adverse consequences of suppression measures.

## Background

In 2020, the outbreak and spread of COVID-19 led many governments around the world to adopt suppression measures, including lockdowns, bans on public events, and social distancing. Such measures, although effective in containing the spread of the virus [1], may have had unintended consequences for the mental health of the population. Brooks and colleagues performed a rapid review of the literature on the psychological impact of quarantine in previous viral outbreaks and reported several negative psychological outcomes [2]. Three main pathways were involved. First, continuous reports of information about outbreaks in the press and social media were likely to increase stress, anxiety, and fear of the disease and its consequences among the population. The confinement period due to COVID-19 occurred in the context of an unprecedented pandemic, a crisis affecting the entire world [3]. Anxiety may have been further increased by the dissemination of verified and unverified information about the consequences and spread of the outbreak [4–6]. Anxiety and stress were likely to be even greater among those affected by the disease at home, those at risk of being affected, and those who had a relative or a close friend outside the home who was affected [3].

Second, the suppression measures deliberately led to a reduction in social contact, social activity, and social support, dramatically changing the social lives of individuals. Limiting social contact affects negatively the mental health of the general population, as evidenced in previous studies [7, 8]. Such limitations have various possible consequences; confinement is likely to increase feelings of stress among individuals by limiting both access to public and open spaces and contact with people outside the home [9, 10]. Confinement also lead to greater social exclusion, loneliness, reduced social support, and an increase in alcohol and substance use, all of which are key risk factors for poor mental health and suicidal behaviour [11–15].

Third, the spread of COVID-19 and suppression measures may increase stress by affecting labour conditions [16]. The workload of some, who were not employed in essential sectors, decreased, while the workload of others, such as health care professionals, increased [17, 18]. There was also an increase in teleworking for many people employed in services [19]. The closure of schools also led to children being stuck at home with their parents. Children were particularly vulnerable to confinement [20], while parents had to combine the management of their professional activities with coping with children at home [21, 22]. Many people also feared possible long-term consequences of the reduction in activity, particularly for employment and for their income [23].

Finally, the COVID-19 outbreak and subsequent policy measures may not affect all sociodemographic groups in the same way. They were likely to affect the social and mental health of some groups of the population which are more vulnerable to the three pathways mentioned above, including women [7, 24] and people who were already physically, mentally, or socially vulnerable prior to the outbreak [25–27]. The main aim of this paper, therefore, was to investigate the risk of psychological distress that may be associated with the COVID-19 pandemic and subsequent confinement measures, particularly during the early days of confinement, in order to measure the short-term effects of the pandemic and the subsequent measures. In light of the previous results reported in the literature, three main research questions were addressed: (a) How did the level of psychological distress at the start of the lockdown period compare with the level of psychological distress usually measured in the general population? (b) Which health, social, and economic conditions predicted psychological distress at the beginning of the confinement period? (c) Was the risk of psychological distress associated with the duration of the lockdown?

Our study complements previous studies in several ways: we compare our results to a dataset from a pre-COVID19 national survey, helping to shed light on the changes, at populational level, associated with the pandemic and the accompanying measures. We also attempt to disentangle the different pathways involved and we compare the level of different symptoms in a pre-COVID19 period with the level of symptoms at the beginning of the lockdown. Finally, Belgium is an interesting case study as it has been among the countries worst hit by the COVID-19 pandemic (see below).

## Methods

### Setting

Belgium has been hit badly by the COVID-19 pandemic. The epidemiological outcomes have been poor: it has one of the highest numbers of deaths per inhabitant and the situation in nursing homes is critical [28]. It was one of the first European countries to implement suppression and nation-wide lockdown measures, including the closure of all schools and higher education institutions. Later, Belgium also pioneered limiting the size of the household social bubble.

### Design and data

We carried out an online survey of the general population in Belgium. This survey design strategy was chosen because movement of the population was restricted and we wanted to quickly evaluate the risk of psychological distress at the very beginning of the lockdown. The survey was widely publicised and disseminated through social media and the main national newspapers and was advertised on the radio and on TV. In Belgium, lockdown measures were implemented from 18 March 2020. The survey was open from 20 March to 9 April 2020. During that period, 27,857 individuals clicked on the web survey and 21,734 agreed to complete it. After excluding responses with missing data, we were left with 20,792 valid responses. The survey was designed to allow comparison with the level of psychological distress found in the Belgian population under normal conditions. To that end, we obtained the most recent such survey, the Belgian Health Interview Survey of 2018 (*n* = 7793), hereafter BE.HIS2018 [29, 30]. This survey, which has been carried out every 4–5 years since 1997, assesses the health status of the population and its social and behavioural determinants, using a representative sample from each of the three Belgian Regions (Flanders, Wallonia, and Brussels).

### Measures

The scales were selected with particular emphasis on validated, short, and population-level scales that had been used in other health surveys, including the pre-COVID-19 BE.HIS, and were already translated and validated in French, Dutch, and English. The primary outcome, psychological distress, was measured with the GHQ-12 as in the BE.HIS. GHQ-12 is a 12-item scale of common mental disorders [31] that displays good psychometric properties, with a Chronbach’s alpha score of 0.90 on the Likert scale [32, 33]. We used the GHQ scoring method, which returns a continuous score ranging from 0 to 12, with a score of 4 or more indicating the likelihood of a mental disorder [33].

We explored health, social, and occupational risk factors. Health risk factors were related to the direct or indirect exposure of the population to the COVID-19 outbreak and to subjection to the subsequent lockdown measures. Exposure was assessed using an index that was constructed on the basis of eight dichotomous (yes/no) questions about proven (tested or diagnosed) or suspected COVID-19 infection of the respondent and/or of someone living with the respondent and/or of a relative or acquaintance. The COVID-19 exposure index ranged from 0 (low exposure) to 8 (high exposure). We also calculated the length of time for which respondents had been subjected to the lockdown measures, using the number of days between 18 March 2020 and the day of completion of the questionnaire.

Social risk factors were related to social activity and support. The volume of social activity was assessed using an adaptation of the Social Participation Measure (SPM), an adaptation that was developed as part of the Common Cold Project [34]. Respondents were asked about the frequency of six types of social activity during a normal week, before and after the start of the lockdown period. The score for change in social activity between a normal week and the first week of the lockdown period ranged from − 18 (considerable increase in activity) to 18 (considerable decrease in activity). Social support was assessed using the 3-item Oslo Social Support Scale, which returns a score ranging from 3 (poor social support) to 14 (strong social support). The social support scores were categorised into three groups (3 to 8: weak; 9 to 11: moderate; 12 to 14: strong social support) [35]. Social isolation was measured using the Short Loneliness Scale (LON), ranging from 3 (low level of loneliness) to 12 (high level of loneliness) [36].

Occupational risk factors were related to changes in occupational status, workload, and income. Respondents were asked whether they had experienced changes in their income, employment status, and/or working conditions (such as increased teleworking) following the COVID-19 outbreak and lockdown measures. Finally, socio-demographics (age, gender, occupational status, and educational status) and items allowing the identification of specific vulnerable subgroups (household composition, profession, and previous history of long-term illness) were also requested and included as control variables. The full questionnaire is available online in French, Dutch, and English (www.uclouvain.be/covidandI).

### Ethical review

Belgian Law does not require approval by an ethics committee for an online survey of the general population. The study is, however covered by privacy regulations. Participants were provided with all legal information relating to consent. All information related to respondents’ consent and the GDPR is available on request.

### Statistical analysis

The statistical analysis was a three-step process. First, we computed the level of psychological distress by age and gender group. Then, we performed linear and logistic regressions in order to examine the association between psychological distress and the independent covarites (exposure to COVID-19, lockdown measures, social and labour conditions), controlling for socio-demographic characteristics and the existence of a previous long-term illness. Finally, we assessed the risk of psychological distress associated with COVID-19 and subsequent lockdown measures by comparing the ratios found in our sample with those found in a pre-COVID-19 benchmark, the BE.HIS2018 sample. As the composition of the two samples differed, the rate of psychological distress in the two samples was calculated conditioning for age, gender, level of education, and employment status using a conditional logistic regression. We also included the social support score in order to control for the potential bias affecting those with a lower level of social support, who may have been more likely to participate in the survey. We bootstrapped 1000 samples, stratified on the three-way national distribution for age group, gender, and level of education, and calculated a 95% bootstrapped confidence interval using the percentile method [37]. Aside from selection biases on observable characteristics, such as gender, age, and education, heterogeneity of unobserved variables may also have affected the measurements. In particular, people who felt a sense of unease due to the COVID-19 pandemic or the lockdown measures might be overrepresented in comparison with the general population, in an online survey. In order to estimate the direction and magnitude of this possible bias, we examined the effect of a well-known risk factor for psychological distress that was available both in our sample and in the BE.HIS2018 sample, i.e. the score on the Oslo Social Support Scale. If our sample was too sensitive to unobserved features, the odds ratio would be greater (in absolute value) in our sample than in the BE.HIS2018 sample. We therefore tested this hypothesis by regressing the Oslo Social Support Scale on psychological distress, controlling for the other socio-demographic variables, and we compared the results of the two samples. Steps 2 and 3 of the analysis were weighted to match the European Standard Population. All statistical analyses were performed using SAS 9.3 for Linux.

## Results

### Sociodemographic characteristics and level of psychological distress in the study sample

**Fig. 1:**
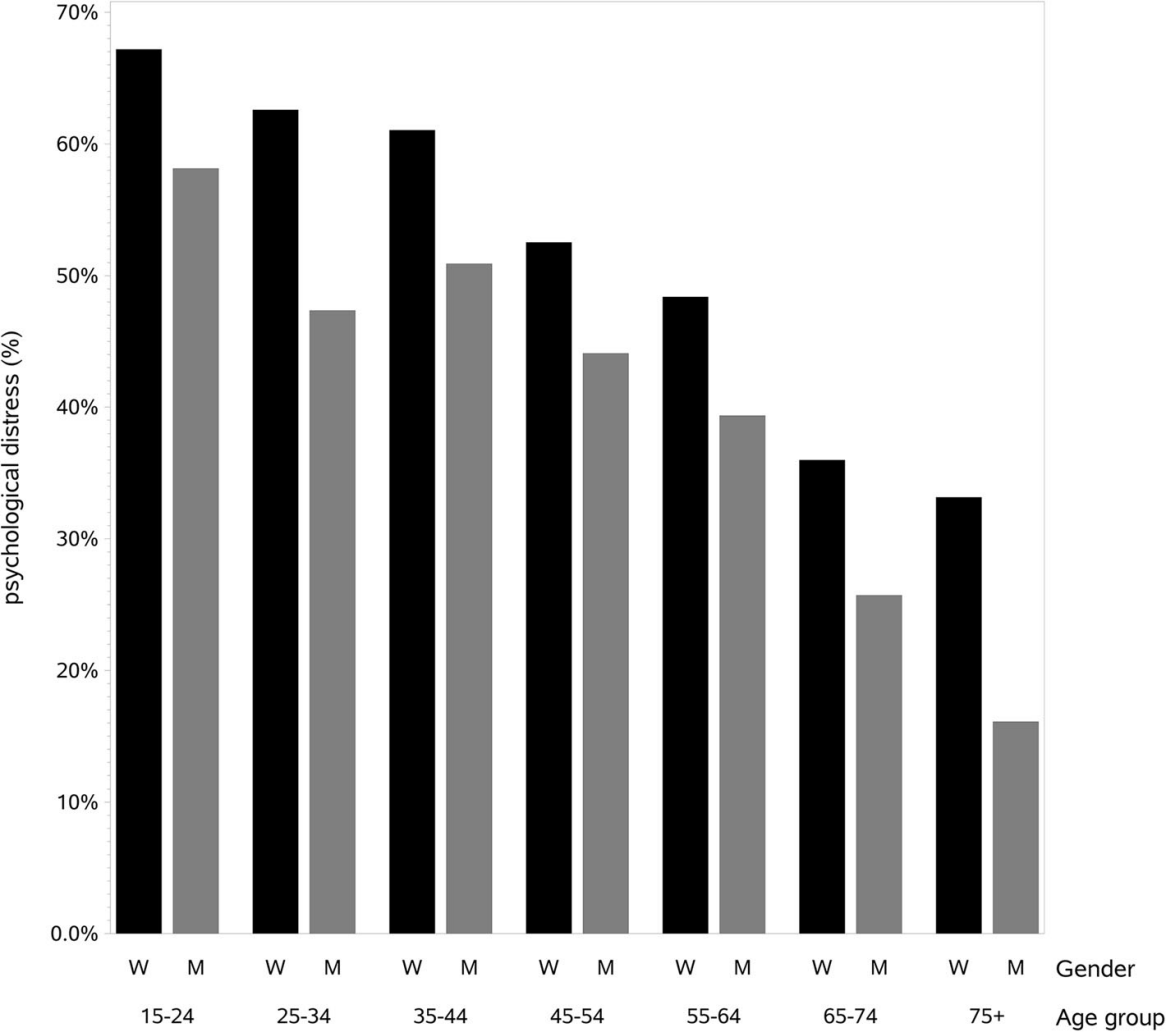
Level of psychological distress in the study sample (March – April 2020, during the COVID-19 lockdown period) by age and gender group: percentage, weighted analysis

The sample is described in Table 1. More than half of the respondents (52.9%) had experienced psychological distress after less than a week of confinement on average (5.6 days). Figure 1 displays the proportion of respondents who experienced psychological distress by age and gender groups: black (for women) and grey (for men). In all age groups, women were at greater risk of psychological distress than men. Psychological distress decreased linearly with age: younger females were almost twice as likely to report psychological distress as older females.

**Table 1:** Descriptive statistics of the study sample, unweighted

	March – April 2020^a^ *N* = 20,792 % (or mean)
Age (mean, std)	43.6 (14.9)
Gender:	72.4
Women	
Men	27.6
Education:	
Secondary or lower	13.6
Higher	79.5
Other	6.9
Social support:	29.4
Weak	
Moderate	47.8
Strong	22.8
Psychological distress:	47.1
No	
Yes	52.9
GHQ – 12 score (mean, std)	4.5 (3.5)
No. of days of confinement (mean, std)	5.6 (4.9)

^a^COVID-19 pandemic study carried out in Belgium, March – April 2020

The risk factors of psychological distress are presented in Table 2: Model 1 includes all the variables displayed in the table, while Model 2 controls for age group, gender, level of education, and the presence of a long-term illness. Individuals who were exposed to COVID-19 were more likely to experience psychological distress (Table 2). In Model 1, each additional point of exposure to the illness was associated with a significant increase in psychological distress (OR = 1.17, *p* < .001). A greater decrease in social activity (OR = 1.11, *p* < .001), a higher level of loneliness (OR = 1.45, *p* < .001), and a lower level of social support (OR = 0.88, *p* < .001) were also associated with greater likelihood of psychological distress. A change in occupational status was also associated with a greater likelihood of psychological distress, including for those who were teleworking more (OR = 1.35, *p* < .001). Likewise, those who had experienced an increase in their workload during lockdown were found to be at greater risk of psychological distress than those who had not experienced workload changes (OR = 2.11, *p* < .001). The results were similar in Model 2, in which confounders were included. In general, the estimates remained significant and of similar magnitude. The signs and the magnitude of the results were also similar to the continuous score of psychological distress.

**Table 2:** Risk factors of categorical and continuous psychological distress: odds ratio and beta from the logistic and linear regressions, weighted analysis^c^

			*Psychological distress*											
	*Mean (std)* *or %*		*Categorical distress*^a^						*Continuous score of distress*^a^					
			*Model 1*^b^			*Model 2*^b^			*Model 1*^b^			*Model 2*^b^		
*Covariates*			*OR*	*95%CI*	*p*	*OR*	*95%CI*	*p*	*Beta*	*95%CI*	*p*	*Beta*	*95%CI*	*p*
Exposure to COVID-19 (no., 0 to 8)	0.94 (1.3)		1.17	1.14,1.20	<.001	1.15	1.12,1.18	<.001	0.17	0.14, 0.21	<.001	0.14	0.11, 0.17	<.001
Duration of lockdown (days, 1 to 21)	5.2 (4.9)		1.03	1.02,1.04	<.001	1.03	1.02,1.03	<.001	0.09	0.08, 0.10	<.001	0.09	0.08, 0.10	<.001
Decrease in social activity (score, −13 to 17)	3.8 (2.9)		1.11	1.10,1.13	<.001	1.10	1.08,1.11	<.001	0.16	0.14, 0.17	<.001	0.13	0.12, 0.15	<.001
Social support (score, 3 to 14)	9.1 (2.4)		0.88	0.87,0.90	<.001	0.87	0.85,0.88	<.001	−0.20	−0.22,-0.18	<.001	−0.20	− 0.22,-0.18	<.001
Loneliness (score, 3 to 12)	6.1 (2.7)		1.45	1.42,1.47	<.001	1.46	1.44,1.49	<.001	0.65	0.63, 0.67	<.001	0.62	0.60, 0.64	<.001
**Change in occupational status (ref = no change):**														
Lost job	1.3		5.24	3.57, 7.69	<.001	4.55	3.08, 6.71	<.001	1.53	1.20, 1.86	<.001	1.26	0.93, 1.58	<.001
Stopped working	14.0		1.08	0.95, 1.22	0.239	1.02	0.90, 1.16	0.775	0.41	0.26, 0.56	<.001	0.29	0.14, 0.45	<.001
More teleworking	47.7		1.35	1.23, 1.49	<.001	1.00	0.90, 1.11	0.950	0.58	0.46, 0.70	<.001	0.22	0.10, 0.35	<.001
More time in workplace	4.2		0.71	0.59, 0.87	<.001	0.54	0.44, 0.66	<.001	−0.42	−0.66,-0.17	<.001	−0.73	−0.97,-0.49	<.001
Other	14.2		1.24	1.11, 1.39	<.001	1.32	1.18, 1.49	<.001	0.51	0.37, 0.65	<.001	0.52	0.38, 0.66	<.001
**Income (ref = no change):**														
Increase	0.5		0.43	0.26, 0.69	<.001	0.43	0.26, 0.71	<.001	−1.01	−1.49,-0.53	<.001	−1.08	−1.55,-0.61	<.001
Decrease	19.3		1.45	1.31, 1.60	<.001	1.48	1.34, 1.64	<.001	0.54	0.42, 0.67	<.001	0.53	0.41, 0.66	<.001
**Workload (ref = no change):**														
Increase	21.3		2.11	1.90, 2.33	<.001	1.87	1.68, 2.08	<.001	0.85	0.72, 0.98	<.001	0.63	0.50, 0.76	<.001
Decrease	40.6		1.69	1.54, 1.85	<.001	1.43	1.30, 1.57	<.001	0.63	0.52, 0.74	<.001	0.37	0.26, 0.48	<.001

^a^ Categorical distress refers to GHQ12 > =4; continuous distress refers to the total score

^b^Model 1 is controlled for all variables displayed in the table; Model 2 is additionally controlled for age group, gender, educational level, and presence of long-term illness

^c^Weighted according to a Standard European Population

### Comparison of the level of psychological distress with a pre-COVID-19 study

**Fig. 2:**
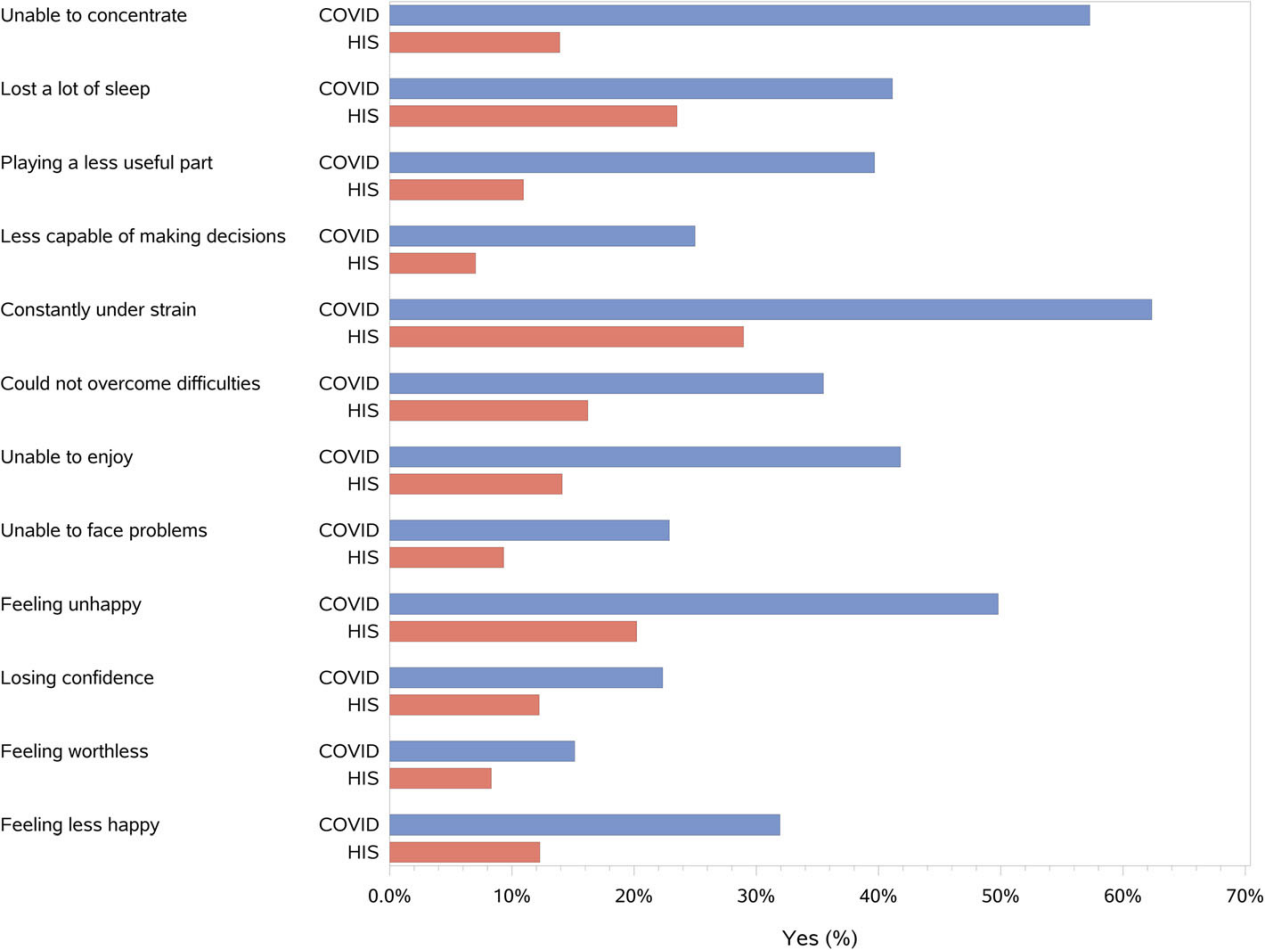
GHQ-12 items, percentages from the study sample (March – April 2020, during the COVID-19 lockdown period) and the BE.HIS2018 sample, weighted sample

Figure 2 displays the differences between the BE.HIS2018 samples (light grey) and the study samples (dark grey) for each of the GHQ-12 items. Three items stood out as showing a considerable increase: 57% of the respondents in the study sample were less able to concentrate than usual (as against 14% in the BE.HIS2018 sample), 40% declared that they felt less useful than usual (as against 11% in the BE.HIS2018 sample), and 62% felt constantly under strain (as against 29% in the BE.HIS2018 sample).

When comparing the rates of psychological distress in the study and in the BE.HIS2018 samples, we observed that the proportion of people who experienced psychological distress was much higher in the study sample (52.8%) than in the BE.HIS2018 sample (18.3%). The bootstrapped mean percentage of psychological distress was 48.9% (95%BC: 48.3, 49.6). The unconditional ratio of the study sample compared to the BE.HIS2018 sample was 2.92, while the conditional rate ratio was 2.3 (95%CI: 2.16–2.45). Finally, we found that a higher level of social support, as measured with the Oslo Social Support Scale, was associated with a lower risk of psychological distress in our sample (OR = 0.82, 95%CI: 0.81–0.83), but with a smaller effect size than in the BE.HIS2018 sample (OR = 0.75, 95%CI: 0.73–0.77), a difference that was statistically significant (*p* < 0.001).

## Discussion

### Main findings

From the first week of lockdown, half the respondents displayed psychological distress, with women and young people displaying the highest levels of psychological distress. A longer period of lockdown, a lower level of social support, a greater reduction in social activity, changes in working conditions, and a higher level of exposure to COVID-19 were all associated with greater risk of psychological distress. Our study is original in that it provides a comparison with a pre-COVID-19 study. That comparison suggests that the COVID-19 pandemic and subsequent confinement measures have led to a more than twofold increase the level of psychological distress in the Belgian population compared to normal levels, as measured in the Belgian Health Interview Survey carried out in 2018.

#### Consistency with previous studies and interpretation of findings

The level of psychological distress measured in our study is quite similar to the results of recent studies carried out in China [38, 39], the USA [40], and other European countries [41–45]. The difference in psychological distress before and after the lockdown is also similar to the findings of a study in the UK which found that distress increased from 19 to 27% [41] and to those of a study in the USA which found that distress increased from 4 to 14% [40]. Another international study that compared eight countries across four continents found that 30.2% of the respondents displayed symptoms of generalised anxiety disorder or major depression [6]. Our findings are also in line with those of Brooks and colleagues: some studies included in that review showed a two- to threefold increase in the psychological distress experienced by those being quarantined, compared to the general population. The finding that women were at greater risk of psychological distress than men is also consistent with previous studies [2, 6, 38, 39]. The downward trend according to age is an unsettled matter in the literature. A greater risk of psychological distress among young people was also reported in the international study mentioned above [6] and in a Chinese study [46]. This is surprising, however, given that older age groups are at greatest risk of mortality from COVID-19. Furthermore, the use of social media, which younger people are generally believed to be more comfortable with, partially compensates for social distancing. One possible explanation for this finding is that confinement measures have a particularly strong (social, occupational, and psychological) impact on younger people, particularly in the early days of lockdown. Older people usually have limited social capital and less diverse social and professional activities than younger people and may, therefore, be less affected by the confinement measures. This interpretation is supported by our analyses, which indicate that this age trend vanished once changes in professional and social activities were factored in.

The finding that greater risk of psychological distress was associated with lockdown duration is consistent with a study carried out in Toronto during the SARS epidemic, which found that people who had spent longer in quarantine were at greater risk of PTSD [11]. It is also consistent with a study carried out in Flanders which found that fear of loneliness was more widespread at the beginning of the lockdown than later on [45]. We cannot, however, dismiss the possibility of a selection bias in our study: people who completed the survey later on may have experienced more mental health issues or risk factors than those who completed the survey earlier. Further longitudinal studies would help to ascertain the effect of the duration of lockdown. The role of health, social, and occupational determinants of psychological distress is consistent with a large body of research in social epidemiology [47–51]. The specific context of this study, however, allows us to add to the literature the finding that short-term changes in social activity, social support, and working conditions resulting from suppression measures have an immediate effect on mental health. One critical aspect is loneliness, which has a major influence on psychological distress. This may signal the loss of a sense of belonging, a key mechanism in the link between social capital, psychological health [47], and physical health [52]. Detailed examination of the GHQ-12 items indicated that there was a 29% increase in the proportion of individuals who felt they were playing a less useful role in life. This is a critical factor during the COVID-19 pandemic. Being confined at home and not being able to carry out personal and professional activities may strengthen that feeling, along with perceived powerlessness to stem the pandemic [53]. That sense of usefulness, therefore, could be targeted by proper intervention. One possibility could be to emphasise the role each individual can play in the fight against the spread of COVID-19 and in taking care of others [54].

### Limitations

Despite the high number of responses received, the main limitation of this study is a selection bias. The whole population was invited to participate in an online survey and those who responded, especially during the early days of lockdown, were those who wanted to have a voice. It is very likely, therefore, that the proportion of people who felt a sense of unease due to the pandemic and confinement measures was high among the respondents. We have indicated that women, more highly educated people, and younger people were overrepresented in the respondent sample; also, because of this sampling selection, it is likely that we underestimated the proportion of those with a more vulnerable occupational status. The effects of this selection bias are, however, unclear. The higher proportion of women makes it more likely that the risk of psychological distress was overestimated, as several studies have indicated that psychological distress is generally greater among women. Likewise, psychological distress tended to be greater in younger age groups in the Belgian Health Interview Survey than it was reported to be in other studies [55], even if the pattern whereby psychological distress decreases according to age was less clear than in our study [30]. By contrast, psychological distress was found to be greater among less educated people than among more highly educated people in the Belgian Health Interview Survey [30]. This suggests that the proportion of highly educated people in our sample may have led to an underestimation of psychological distress. Furthermore, the use of an online survey meant that we were unable to reach out to the most vulnerable groups of the population, who had no access to the survey. It is likely that the psychological distress of the population was also underestimated for that reason. It is difficult to separate the impacts of these different factors. The bootstrapped average of psychological distress would indicate that the selection bias led to an overestimation of the psychological distress in the general population. The effect size of the odds ratio in the study and the BE.HIS2018 samples, however, indicates that the magnitude of risk factors was slightly underestimated in our study.

## Conclusions

The short-term health, social, and economic conditions related to the COVID-19 pandemic and subsequent lockdown measures were associated with a worsening of the mental health of the general population in Belgium. The effects were measurable from the very first days of lockdown. The risk of psychological distress increased in accordance with increases in exposure to COVID-19 and duration of confinement. This was one of the first assessments of the mental health effects of the COVID-19 pandemic and confinement measures in Europe to be based on a large population sample. The findings indicate that, from the point of view of mental health, the authorities should limit the duration of lockdown and social distancing measures to a minimum. The authorities should also pay attention to those groups of the population that are most at risk of psychological distress, e.g. women, young people, people who are experiencing changes in their occupational status, and people who are feeling lonely or socially isolated. There might be intergenerational tension, as the mental health burden of lockdown seems to fall most heavily on younger people, even though the elderly are more at risk from COVID-19. Mitigating the impact of lockdown on people’s social and professional lives might be an effective strategy for coping with long periods of lockdown. Further research is needed, however, to evaluate whether the mental effects of COVID-19 and lockdown are sustained over longer periods. Likewise, further research should assess whether these effects are similar, in nature and size, in different countries, particularly by taking into account differences in the intensity of the outbreak and the diversity of the suppression measures implemented in the different countries affected.

## Data Availability

Consent was obtained from the participants on condition that their data would not be shared, as stipulated in the Data Management Plan. A limited set of the data is included in the supporting information section of the paper in order to allow analyses replication.
